# Role of rs454214 in Personality mediated Depression and Subjective Well-being

**DOI:** 10.1038/s41598-020-62486-x

**Published:** 2020-03-30

**Authors:** Binyin Hou, Lei Ji, Zhixuan Chen, Lin An, Naixin Zhang, Decheng Ren, Fan Yuan, Liangjie Liu, Yan Bi, Zhenming Guo, Gaini Ma, Fei Xu, Fengping Yang, Shunying Yu, Zhenghui Yi, Yifeng Xu, Lin He, Chuanxin Liu, Bo Bai, Shaochang Wu, Longyou Zhao, Changqun Cai, Tao Yu, Guang He, Yi Shi, Xingwang Li

**Affiliations:** 1 Bio-X Institutes, Key Laboratory for the Genetics of Developmental and Neuropsychiatric Disorders, Shanghai Jiao Tong University, 1954 Huashan Road, Shanghai, 200030 China; 2 School of Mental Health, Jining Medical University, 16 Hehua Rd, Taibaihu New District, Jining, Shandong 272067 China; 3 Shanghai Key Laboratory of Psychotic Disorders, and Brain Science and Technology Research Center, Shanghai Jiao Tong University, 1954 Huashan Road, Shanghai, 200030 China; 4 Lishui No.2 People’s Hospital, 69 Beihuan Rd, Liandu District, Lishui, Zhejiang 323000 China; 5 Wuhu No.4 People’s Hospital, 1 Xuxiashan Rd, Wuhu, Anhui 241002 China

**Keywords:** Genetic association study, Depression, Psychology

## Abstract

Happiness and depression are interlinked and both heritable, while personality, as an important predictor of them, shares the genetic basis with them. We conjecture that genetic factors of depression can affect both depressive symptoms (DS) and subjective well-being (SWB), while personality traits play important roles in mediating this process. In this study, 878 Han Chinese college freshmen and 384 Han Chinese patients with the major depressive disorder (MDD) were included. SNPs were genotyped using AGENA MassARRAY iPLEX technology and we investigated an important MDD variant rs454214. Correlation, association and mediation analysis were employed, aiming to decipher the complex relationship between SWB, DS, personality traits and the genetic variant. Association study indicated that rs454214 was not only associated with both SWB and DS (P < 0.05), but also possibly linked to MDD. Mediational analysis showed that rs454214 had no direct effect on SWB and DS, but had a significant indirect effect through personality traits, i.e., Extraversion, Neuroticism, Agreeableness and Openness to Experience or SWB, Extraversion, Neuroticism and Agreeableness for DS. This study found a shared genetic basis for happiness and depression; the causal process could be better explained if personality traits are taken as mediating factors.

## Introduction

Subjective well-being(SWB) is a subjective measure of one’s emotion and cognition, consisting of positive and negative affect as well as life satisfaction which stands for happiness^1^. In contrast, depression can be a serious disorder with significantly increased risk of physical and mental disabilities^2,3^. The depressive symptoms (DS) are warning signals for one’s psychological health and if not taken seriously, the individual can easily develop into clinical depression^4^. Happiness and depression are negatively correlated with each other^5^; they both show heritability at a rate from 30% to 40%, according to many twin and family studies^6–8^. Single nucleotide polymorphisms (SNPs) that are significantly associated with them were discovered as well^9,10^.

On the other hand, according to previous studies, personality traits had been widely reported as important predictors of subjective well-being (SWB)^11,12^ and depression^10,13,14^, which can usually be illustrated as a five-factor model (FFM), including Extraversion (E), Neuroticism (N), Conscientiousness (C), Agreeableness (A), and Openness to Experience (O)^15^. Twin studies suggested it with a heritability of nearly 40%^16^ and genome-wide association studies (GWAS) have probed several remarkable associating SNPs^17^.

In addition, correlation was found between personality and SWB as well as depression^10,12,17^ in a genetic level. This complex intrinsic correlation suggested a shared genetic inheritance of happiness and depression and we conjecture that personality traits can play an important role as a mediator.

The purpose of this study was to explore the possible shared genetic basis of SWB and DS through the MDD GWAS-supported SNP^9^ and the mediating role of personality.

## Materials and methods

### Participants

The participants in this study were 878 Han Chinese college freshmen. The mean age of the participants was 18.7 years (SD = 1.15), and 37.5% of the participants were male. Additional information about participant characteristics was given in Table 1. We also recruited 384 Han Chinese patients with MD (175 males and 209 females; mean age 35.4+/−12.18 years, range18–60 years) in this research to as a validation cohort. The MDD diagnosis were conducted by at least two well-trained psychiatrists using the Structured Clinical Interview for DSM-IV (SCID-I)^18^. All of them signed the informed consents and the study was appraised and confirmed by the Ethics Committee of the Jining Medical University and Shanghai human genetic resources ethics committee. All methods were carried out in accordance with relevant guidelines and regulation.

**Table 1:** Demographics and characteristics of the college sample.

Variable	N	%
Gender		
Males	329	37.5
Females	549	62.5
	**Mean**	**SD**
Age	18.70	1.15
Depressive symptoms	13.77	10.88
Positive affect	34.11	7.74
Negative affect	22.22	8.07
Satisfaction with life	20.41	7.28
Oxford happiness	36.18	7.51
Personality		
Extraversion	26.17	6.72
Agreeableness	35.07	5.53
Conscientiousness	29.72	6.14
Neuroticism	23.37	6.12
Openness	34.80	6.14

The freshmen filled the questionnaire including demographic information, assessment of SWB, DS and personality as well as information feedback to the survey (efficacy, understanding, carefulness, significance). Data cleaning and curation was performed based on physical criterion, clustering analysis, feedback questions and questionnaire response time control (2% omitted in total).

### Measurements

Assessment of subjective well-being (SWB) is composed of multiple measuring scales, including Satisfaction With Life Scale (SWLS^19^), the Positive and Negative Affect Scale (PANAS^20^) and Oxford Happiness Questionnaire (OHQ^21^). The final score of subjective well-being was calculated using Principal Component Analysis (PCA).

We measured depressive symptoms (DS) using the revised Chinese version of CES-D (Center for Epidemiologic Studies Depression Scale)^22^. It can be used as a self-report depressive symptomatology measurement for research in the general population. The CES-D scores range from 0 to 60, with the higher score correspond to more serious depression. Criteria: the total score ≤15 is considered as no depression, and the total score of 16~19 is considered as possible depression, and the total score > = 20 indicates definite depression (moderate to severe)^23–25^.

Personality was assessed by the Big Five Inventory (BFI) measurement on the Big Five dimensions including E, N, A, C and O^26^.

Supplementary Table 1 shows some details and scoring scheme of above scales.

### DNA extraction and genotyping

We collected the peripheral venousblood from each freshmen and MD patient and applied Trizol protocol in DNA extractions. The major depressive disorder GWAS-supported variant rs454214 in *TMEM161B*-*MEF2C*^9^ were genotyped by time of flight (MALDI-TOF) mass spectrometer through MassARRAY Analyzer four platform (AGENA, San Diego, CA). All the primers were designed by My-Sequenom online soft-ware Assay Design Suite v2.0. Each tube in the polymerase chain reaction contained 10 ng genomic DNA dissolved in 5 μl buffer.

### Statistical analysis

#### Descriptive statistics

For all analyses, we set statistical significance at *P* value <0.05. Supplementary Table 2 summarizes the R packages we use. Descriptive statistics were calculated using statistical packages in R Studio including the basic information of the sample, the mean and standard deviation of SWB, depression symptoms and personality. T-test was used to compare the groups of different genders.

#### Correlation analysis

In this study, correlation analysis and its heat map were achieved using “Corrplot” package in R Studio. Association analyses were conducted by R package “SNPassoc” with five different genetic models (codominant, dominant, recessive, over-dominant and log-additive models, respectively).

To test whether DS and SWB share the same affecting genetic variant, rs454214, we first analyzed the correlation between DS and SWB, and the correlation between rs454214 and the scores of both SWB and DS. Moreover, we performed another analysis to validate the association between rs454214 and clinical depression (387 cases with MD, 878 controls). Information of allele and genotype frequency were obtained by using the SHEsis webserver (http://analysis.bio-x.cn/myAnalysis.php) and the “SNPassoc” package. The result of Hardy-Weinberg equilibrium test was also generated this way.

#### Mediation analysis

We conjecture that personality play a mediating role in the path from rs454214 genotype to SWB/DS phenotype. To test this hypothesis, we performed another correlation analysis for 5 dimensions of personality with SWB/DS. We also conducted the same analysis for rs454214 and the 5 dimensions of personality.

Furthermore, we tried to verify the mediational models in which personality mediate the relationships between rs454214 and SWB/DS; the R package “semMediation” was employed in this analysis. And we used the “mediation” R package to validate the result of each mediator respectively with bootstrapping of the indirect effect. Bootstrapping was used over alternative tests to avoid Type 1 errors that may arise from non-normal distributions of an indirect effect^27^. It could also provide additional information about percentage of mediating variables explaining the association between X and Y, i.e., the genotype and phenotype.

## Results

### Degree of depressive symptoms and subjective well-being

Descriptions of statistical scales are shown in Table 1. The mean score of CESD is 13.77, belonging to “no depressive symptoms” level (<=15). The degree of satisfaction with life is “Neutral” (20). And the average score of OHQ reach the level of “Rather happy” (4–5). In this study, there shows no significant difference in terms of DS, SWB, and personality scores between males and females (P > 0.05).

After Cattell Scree Test^28^, Parallel Analysis^29^ combined with Kaiser-Harris Criterion^30^ of Principal Component Analysis (PCA), the first principal component was taken as the final measurement score of Subjective Well-being.

### Rs454214 is a shared variant of SWB/DS

**Figure 1:**
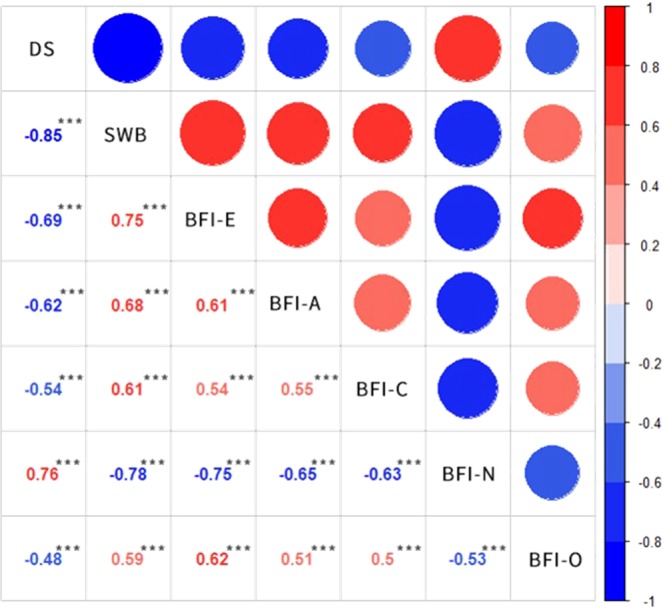
Heat map illustrating correlations between personality traits and depressive symptoms and subjective well-being. The values in the lower triangular matrix is correlation coefficients. In the upper triangular matrix, big circles in dark colors indicates strong correlations. Positive correlations are showed in red while negative correlations are showed in blue. DS: depressive symptoms, SWB: Subjective well-being, BFI-E: Extraversion, BFI-A: Agreeableness, BFI-C: Conscientiousness, BFI-O: Openness to experience, BFI-N: Neuroticism. All correlation coefficients were significant at 0.001 level. *p < 0.05; **p < 0.01; ***p < 0.001.

Correlation analysis showed that SWB was strongly negatively correlated to DS (β = −0.85, P < 0.001, Fig. 1). We tested five genetic models and found significant association between the candidate SNP and SWB/DS in three of them, i.e., codominant, recessive, and overdominant, (*P* < 0.05, Table 2). Codominant model was then excluded due to 95% confidence interval constraint (contains zero). In the overdominant model, TC genotype referred to a higher state of SWB and lower score of DS (compared to TT and CC), while the recessive model indicated that TC and TT genotypes play a more positive role in SWB comparing to the CC genotype.

**Table 2:** Association between rs454214 and subjective well-being as well as depressive symptoms.

Genotype rs454214	N	Mean^a^	SE^a^	β ^a^(95%CI)	p^a^-value	Mean^b^	SE^b^	β ^b^(95% CI)	p^b^-value
**Codominant**									
T/T	279	13.85	0.62	0.00	0.0415^*^	−0.05	0.06	0.00	0.0228^*^
T/C	411	13.02	0.55	−0.83 (−2.48, 0.82)		0.09	0.05	0.14 (−0.01, 0.29)	
C/C	184	15.45	0.82	1.60 (−0.42, 3.62)		−0.13	0.07	−0.08 (−0.27, 0.10)	
**Dominant**									
T/T	279	13.85	0.62	0.00	0.9178	−0.05	0.06	0.00	0.3172
T/C-C/C	595	13.77	0.46	−0.08 (−1.63, 1.47)		0.02	0.04	0.07 (−0.07, 0.21)	
**Recessive**									
T/T-T/C	690	13.36	0.41	0.00	0.0203^*^	0.04	0.04	0.00	0.0414^*^
C/C	184	15.45	0.82	2.09 (0.33, 3.86)		−0.13	0.07	−0.17 (−0.33, −0.01)	
**Overdominant**									
T/T-C/C	463	14.49	0.50	0.00	0.0464^*^	−0.08	0.05	0.00	0.0092^*^
T/C	411	13.02	0.55	−1.47 (−2.91, −0.03)		0.09	0.05	0.18 (0.04,0.31)	
**log-Additive**									
0,1,2				0.64 (−0.36, 1.64)	0.2125			−0.02 (−0.12, 0.07)	0.6127

Note.

^a^Association between rs454214 and depressive symptoms;

^b^Association between rs454214 and subjective well-being. Significant P (<0.05) values are in bold. *p < 0.05.

For validation purpose, we recruited 384 Han Chinese patients with MD to examine whether rs454214 is also associated with clinical depression. It turned out that rs454214 passed the Hardy−Weinberg equilibrium test (HWE, *P* > 0.05) both in MD (case) and college samples (control). The genotype frequency of rs454214 between cases and controls was consistent with the recessive model **(***P* < 0.05) according to five genetic models analyzed by R package “SNPassoc”.

### Correlation between personality, SWB and DS

Correlation analysis indicates that the phenotyes of the five personality traits are correlated with SWB/DS. The Heat map (Fig. 1) illustrates that Subjective Well-being is positively correlated with E, A, C and O, and is negatively associated with N in a significant level of 0.001. On the other side, E, A, C and O are negatively associated with DS, while N is positively associated with DS. Likewise, E, A, C, O, and N have significant inter-correlation relationships with one another (*P* < 0.001).

### Association analysis of personality

After the association study between personality and rs454214 (Supplementary Table 3), different genotypes of rs454214 are connected to all the five aspects of personality, fitting the overdominant model. N and A were also significant in codominant model (*P* < 0.05) but failed to fitting the 95% confidence interval constraint (contains zero).

It showed that the CT genotype of rs454214 is associated with higher score of E, A, O, C, and lower score of N. In the previous two analyses hereinabove, we had found the correlation between personality and SWB/DS as well as rs454214. Altogether, the inter-correlations among the variables provided initial support to the hypothetical conjectured indirect effects.

### Mediation analysis

**Figure 2:**
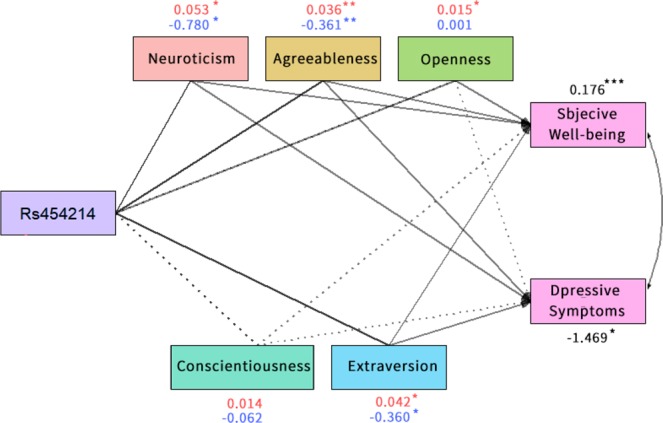
Multi-mediation plot shows indirect effect between rs454214, personality traits, subjective well-being and depressive symptoms. Full lines indicate significant mediated paths in the full model (p < 0.05). Dashed lines indicate insignificant ones (p > 0.05). Estimates of indirect effect on subjective well-being are shown in red. Estimates of indirect effect on depressive symptoms are shown in blue. Estimates of total effect on them are shown in black. *p < 0.05; **p < 0.01; ***p < 0.001.

The multi-mediation analysis and the results in the plot generated by “semMediation” (Fig. 2) revealed the indirect effects of rs454214 on subjective well-being through E, A, O, N (P < 0.001) and those on DS through E, A, and N. Heterozygotes (TC genotype) can act as a protective factor, reducing the level of N and increasing the level of A and E to inhibit depression, and promoted happiness by raising the level of N and suppressing the degree of E, A, and O.

We then tested whether aspects of personality fully or partially mediate the relationship between rs454214 and SWB/DS, by examining whether the direct effect of rs454214 on SWB/DS (controlling for personality) was statistically significant. As demonstrated in Supplementary Fig. 1, the direct effects of rs454214 on subjective well-being and DS are not significant after controlling the impacts of mediators (β = 0.02, 0.09 respectively, *P* > 0.05), supporting an inference of a full mediation.

Supplementary Table 4 shows the verifying result of individual mediation model test by the “Mediation” R package. It is shown that the total effect of each model is significant (*P* < 0.05). We also found that rs454214 facilitated or suppressed SWB/DS through personality (E, A, O, N/E, A, N) with significant *P* value of indirect effect. As zero was not contained in the 95% confidence interval, the conjecture of indirect effect was supported. Direct effect and proportion information can also be found in the Supplementary Table 4. None of the direct effects were significant indicating the full mediation model. Among the five personality aspects, the mediating effect of N explained the highest significant percentage of the total effect (71% for both SWB and DS, *P* < 0.05), followed by E (67% for SWB and 61% for DS, *P* < 0.05) and A (63% for SWB and 59% for DS *P* < 0.05). O could explain 46% of inheritance of SWB due to rs454214 (*P* < 0.05).

## Discussion

The result demonstrated a relatively good situation of psychological health state in the college participants. Whether gender affects SWB and DS was controversial, and in this study, we did not find significant difference between males and females in terms of SWB/DS and personality traits. But it could vary from center to center.

Different from previous studies which measure SWB from a single aspect, multiple scales were adopted to measure SWB for each individual and PCA (principal component analysis) was additionally adopted, according to the SWB model proposed by Diener^1^, making our measurement of SWB more thorough and robust.

We found rs454214 in *TMEM161B*-*MEF2C*^9^ to be a shared genetic loci of DS and SWB in health college participants. Rs454214 was also linked to clinical depression in patients with MDD. It supports our conjecture that happiness and depression partially share the same genetic mechanism. Adolescent depression is difficult to diagnose and easy to be neglected, making depressive symptoms important warning signals^25^. Our direct replication of the genome-wide significant associations with depressive symptoms in an independent depression sample provided further confirmation for this knowledge. Therefore, cares for the mental health of college students and depression prevention are critically important; previous studies also share this point of view^31,32^.

We found that rs454214 might affect DS and SWB in three genetic models: codominant, recessive, and overdominant models. It showed that the TT genotype was the risk factor of DS while the TC genotype was the protective factor. The superiority of heterozygotes was evident in the result. This inverted U-shaped relation was similar to the findings in sickle-cell disease and cognitive control processes. It was recently found that homozygotes of βS sickle mutation can lead to sickle-cell disease while heterozygotes contribute to higher protection^33^. The heterozygotes of CHRNA4 polymorphism (rs1044396) showed higher activity during cognitive engagement than the homozygotes in the cingulo-opercular (CO) network^34^.

Rs454214 is located in the upstream of *MEF2C* gene, which is a transcription factor regulating neuron maturation processes^35–37^. It can promote cognition (learning and memory) through negative feedback regulation that inhibits the over-generation of excitatory synapses and rebuilds the neural network continuously. It is highly expressed in the frontal cortex, entorhinal cortex, dentate gyrus, and amygdala of CNS^38^. These brain regions play essential roles in reward, disgust, stress, and emotional control, which may lead to the phenotype of depression and happiness^39,40^. Previous studies also found strong correlation between personality traits and these brain regions^41,42^, so it is no surprise for us to identify that rs454214 is associated with personality traits and that it affects happiness and depression.

As we found a pairwise causal relationship between rs454214, personality traits and DS/SWB combining with results of the previous studies, mediation analysis was suitable for this study to explain the effect of rs454214, personality traits on DS/SWB. Recent studies reported that Bayesian Structural Equation Model (BSEM) can become an equivalent model for the general Mediated Model we use^43^. Different from standard practice relies on frequentist methods (Bootstrapping and Sobel method) we use, alternative Bayesian approach employs Monte Carlo method and is easier to work with complex models^44^.

Besides suggesting the mediating role of personality, the result of mediation analysis also revealed the differences between different personality traits. In previous research on the relationship between personality, subjective well-being and depression, N and E were regarded as the most prominent personality trait^45,46^. Neuroticism is a dimension of personality which often presents negative emotions such as depression and is negatively correlated with happiness^12,13^. E on the other hand, showed opposite pattern as N^14^, which were again confirmed in our result of correlation analysis and mediation analysis. Furthermore, N and E accounted for the highest proportion of mediating effect between genetic variant (rs454214) and SWB, as well as DS, indicating the importance of E and N in the inheritance of happiness and depression.

In contrast, most of the studies found that A, C, and O had little or no effect on happiness and depression^12,13,47^. In our study however, we found that A played an important role in the genetic of subjective well-being and depressive symptoms. This discovery was supported by another previous study with Han Chinese sample^48^. It illustrated the complexity of happiness and depression that the influence of personality on them can be affected by other factors such as ethnic background, cultural differences, life events. In addition, O also mediated the inheritance of happiness to certain extent. Previous study indicated that this may be achieved by affecting the efficiency of information processing within the brain^49^.

One of the limitations of this study is the relatively small size and that only one SNP was intensively exploited for limited candidate SNP pool. More genetic candidates of depression and happiness will be included with larger sample size in the future work. In addition, the Mediated Model we used was relative simple and only included personality so far. Therefore, even the present model partly demonstrates the important role of personality in the inherited process of DS and SWB, it inevitably excluded effect from some other underlying factors and may not accurately estimating the whole complex genetic and psychological network. More factors such as social support, which is believed to play a more complex role in the existing multi-mediation model^50,51^, learning and memory,which can be influenced by gene MEF2C through regulating synaptic transmission^35,52^, will also be included in future work. With the increase of considering factors, machine learning models like BSEM (Bayesian network for continuous variable^43^) and mixed graphical models (both continuous and discrete variables^53^) may be fit for the construction of complex network. We will also conduct longitudinal research to track how heredity, personality and other factors influence happiness and depression as participants progress through college study.

In conclusion, this study found a relatively comforting situation of the mental health condition of college participants and found the major depressive disorder GWAS-supported variant rs454214 was a shared genetic variant of the subjective well-being and depressive symptoms, which is highly possibly mediated by personality traits. These findings add supporting evidences to the genetic mechanisms of happiness and depression and implies that more attentions need to be paid to college students’ mental health from the perspective of cognition and personality so that early prevention of depression can be achieved.

## Supplementary information

Supplementary information.

## References

[1] Diener E (2000). Subjective well-being. The science of happiness and a proposal for a national index. American Psychologist.

[2] Dunn EC (2015). Genetic determinants of depression: recent findings and future directions. Harvard Review of Psychiatry.

[3] Saba M (2007). Depression, chronic diseases, and decrements in health: results from the World Health Surveys. Lancet.

[4] Katja B, Susanne K, Pine DS (2009). Anxiety and anxiety disorders in children and adolescents: developmental issues and implications for DSM-V. Psychiatric Clinics of North America.

[5] Beijsterveldt V, Toos CEM (2013). Exploring the Association Between Well-Being and Psychopathology in;Adolescents. Behavior Genetics.

[6] Nes RB, Czajkowski NK (2010). Family matters: happiness in nuclear families and twins. Behavior Genetics.

[7] Nes RB, Røysamb E, Tambs K, Harris JR, Reichborn-Kjennerud T (2006). Subjective well-being: genetic and environmental contributions to stability and change. Psychological Medicine.

[8] Sullivan PF, Neale MC, Kendler KS (2000). Genetic epidemiology of major depression: review and meta-analysis. The American Journal of Psychiatry.

[9] Hyde CL (2016). Identification of 15 genetic loci associated with risk of major depression in individuals of European descent. Nature Genetics.

[10] Okbay A (2016). Genetic variants associated with subjective well-being, depressive symptoms, and neuroticism identified through genome-wide analyses. Nature Genetics.

[11] Deneve KM, Cooper H (1998). The happy personality: a meta-analysis of 137 personality traits and subjective well-being. Psychological Bulletin.

[12] Weiss A, Bates TC, Luciano M (2008). Happiness Is a Personal(ity) Thing: The Genetics of Personality and Well-Being in a Representative Sample. Psychological Science.

[13] Kendler KS, Myers J (2010). The genetic and environmental relationship between major depression and the five-factor model of personality. Psychological Medicine.

[14] Luciano M (2017). Association analysis in over 329,000 individuals identifies 116 independent variants influencing neuroticism. Nature Genetics.

[15] Digman JM (1990). Personality Structure: Emergence of the Five-Factor Model. Annual Review of Psychology.

[16] Tena V, Denis B (2015). Heritability of personality: A meta-analysis of behavior genetic studies. Psychological Bulletin.

[17] Lo MT (2017). Genome-wide analyses for personality traits identify six genomic loci and show correlations with psychiatric disorders. Nature Genetics.

[18] Us, A. P. A. W. D. *Diagnostic and statistical manual of mental disorders (4th ed.)*. (2013).

[19] Diener E, Emmons RA, Larsen RJ, Griffin S (1985). The Satisfaction with Life Scale. Journal of Personality Assessment.

[20] Watson D, Clark LA, Tellegen A (1988). Development and validation of brief measures of positive and negative affect: the PANAS scales. Journal of Personality and Social Psychology.

[21] Hills P, Argyle M (2002). The Oxford Happiness Questionnaire: a compact scale for the measurement of psychological well-being. Personality & Individual Differences.

[22] Radloff LS (1977). The CES-D scale. Applied Psychological Measurement.

[23] Herrman H (2002). Longitudinal investigation of depression outcomes in primary care in six countries: the LIDO study. Functional status, health service use and treatment of people with depressive symptoms. Psychological Medicine.

[24] University, G. Z. Shanghai & PRChina. Research on Depression of Postgraduates in a Certain Famous University in Shanghai. *China Journal of Health Psychology* (2009).

[25] Hauenstein EJ (2012). Depression in Adolescence. Journal of Obstetric Gynecologic & Neonatal Nursing Jognn.

[26] John, O. P. & Srivastava, S. The Big-Five Trait Taxonomy: History, Measurement, and Theoretical Perspectives (1999).

[27] Mackinnon DP, Lockwood CM, Williams J (2004). Confidence Limits for the Indirect Effect: Distribution of the Product and Resampling Methods. Multivariate Behavioral Research.

[28] Cattell RB (1966). The Scree Test For The Number Of Factors. Multivariate Behavioral Research.

[29] Hayton JC, Allen DG, Scarpello V (2004). Factor retention decisions in exploratory factor analysis: a tutorial on parallel analysis. Organizational Research Methods.

[30] Kabacoff, R. *R in Action*. (2011).

[31] Medicine, I. O. Preventing mental, emotional and behavioral disorders among young people: progress and possibilities. *Washington DC: National Academies Press*. (2009). 20662125

[32] Lawton A, Moghraby OS (2015). Depression in children and young people: identification and management in primary, community and secondary care (NICE guideline CG28). Archives of Disease in Childhood Education & Practice Edition.

[33] Sadaghiani S (2017). Overdominant Effect of a CHRNA4 Polymorphism on Cingulo-Opercular Network Activity and Cognitive Control. The Journal of Neuroscience: the Official Journal of the Society for Neuroscience.

[34] Laval G (2019). Recent Adaptive Acquisition by African Rainforest Hunter-Gatherers of the Late Pleistocene Sickle-Cell Mutation Suggests Past Differences in Malaria Exposure. American Journal of Human Genetics.

[35] Mitchell, A. C. *et al*. MEF2C transcription factor is associated with the genetic and epigenetic risk architecture of schizophrenia and improves cognition in mice. *Molecular Psychiatry* (2017). 10.1038/mp.2016.254 PMC5966823 28115742

[36] Barbosa AC (2008). MEF2C, a transcription factor that facilitates learning and memory by negative regulation of synapse numbers and function. Proceedings of the National Academy of Sciences of the United States of America.

[37] Lyons, G. E., Micales, B. K., Schwarz, J., Martin, J. F. & Olson, E. N. Expression of mef2 genes in the mouse central nervous system suggests a role in neuronal maturation. *Journal of Neuroscience the Official Journal of the Society for Neuroscience* **15**, 5727–5738 (1995). 10.1523/JNEUROSCI.15-08-05727.1995 PMC6577647 7643214

[38] Leifer D, Golden J, Kowall NW (1997). Myocyte-specific enhancer binding factor 2C expression in human brain development. Journal of Molecular Neuroscience.

[39] Phelps EA, Ledoux JE (2005). Contributions of the amygdala to emotion processing: from animal models to human behavior. Neuron.

[40] Davidson RJ (2002). Anxiety and affective style: role of prefrontal cortex and amygdala. Biological Psychiatry.

[41] Lewis GJ (2014). Heritable influences on amygdala and orbitofrontal cortex contribute to genetic variation in core dimensions of personality. Neuroimage.

[42] Meyer LA, Kolachana BB (2009). Genetic variants in AVPR1A linked to autism predict amygdala activation and personality traits in healthy humans. Molecular Psychiatry.

[43] Palomo, J., Dunson, D. B. & Bollen, K. Bayesian Structural Equation Modeling-8. 163–188 (2007).

[44] Tofighi D, MacKinnon DP (2016). Monte Carlo Confidence Intervals for Complex Functions of Indirect Effects. Structural Equation Modeling A Multidisciplinary Journal.

[45] Lyubomirsky S, Sheldon KM, Schkade D (2005). Pursuing Happiness: The Architecture of Sustainable Change. Review of General Psychology.

[46] Lykken DTA (2010). Happiness Is a Stochastic Phenomenon. Psychological Science.

[47] Roman K, Wakiza G, Frank S, David W (2010). Linking “big” personality traits to anxiety, depressive, and substance use disorders: a meta-analysis. Psychological Bulletin.

[48] Liwei Zhang E (2005). Prediction of Chinese life satisfaction: Contribution of collective self-steem. International Journal of Psychology.

[49] Beaty RE (2016). Personality and complex brain networks: The role of openness to experience in default network efficiency. Human Brain Mapping.

[50] Tan CS, Krishnan SAP, Lee Q-W (2017). The Role of Self-Esteem and Social Support in the Relationship between Extraversion and Happiness: a Serial Mediation Model. Current Psychology.

[51] Lauriola M, Iani L (2017). Personality, Positivity and Happiness: A Mediation Analysis Using a Bifactor Model. Journal of Happiness Studies.

[52] Adachi M, Lin PY, Pranav H, Monteggia LM (2016). Postnatal Loss of Mef2c Results in Dissociation of Effects on Synapse Number and Learning and Memory. Biological Psychiatry.

[53] Haslbeck, J. M. B. & Waldorp, L. J. MGM: Estimating Time-Varying Mixed Graphical Models in High-Dimensional Data. *Statistics* (2015).

