# Supplementary material for: Role of rs454214 in Personality mediated Depression and Subjective Well-being
Source: Sci Rep. 2020 Mar 30;10:5702. doi: 10.1038/s41598-020-62486-x (PMC7105480; doi:10.1038/s41598-020-62486-x)
Supplement: Supplementary file 1 — Supplementary information. [file 41598_2020_62486_MOESM1_ESM.docx]

Role of rs454214 in Personality mediated Depression and Subjective Well-being

Binyin Hou^a,c^ , Lei Ji^a,c^ , Zhixuan Chen^a,c^ , Lin An^a,c^ , Naixin Zhang^a,c^ , Decheng Ren^a,c^ , Fan Yuan^a,c^ , Liangjie Liu^a,c^ , Yan Bi^a,c^ , Zhenming Guo^a,c^ , Gaini Ma^a,c^ , Fei Xu^a,c^ , Fengping Yang^a,c^ , Shunying Yu^c^ , Zhenghui Yi^c^ , Yifeng Xu^c^ , Lin He^a,c^ , Chuanxin Liu^b^ , Bo Bai^b^ , Shaochang Wu^d^ , Longyou Zhao^d^ , Changqun Cai^e^ , Tao Yu^a,c^ , Guang He^a,c*^, Yi Shi^a,c*^ , Xingwang Li^a,c *^

a Bio-X Institutes, Key Laboratory for the Genetics of Developmental and Neuropsychiatric Disorders, Shanghai Jiao Tong University, 1954 Huashan Road, Shanghai 200030, China

b School of Mental Health, Jining Medical University, 16 Hehua Rd, Taibaihu New District, Jining, Shandong 272067, China

c Shanghai Key Laboratory of Psychotic Disorders, and Brain Science and Technology Research Center, Shanghai Jiao Tong University, 1954 Huashan Road, Shanghai 200030, China

d Lishui No.2 People's Hospital, 69 Beihuan Rd, Liandu District, Lishui, Zhejiang 323000, China

e Wuhu No.4 People's Hospital, 1 Xuxiashan Rd, Wuhu, Anhui 241002,China
* Corresponding author at: Bio-X Center, Shanghai Jiao Tong University, 1954 Huashan Road, Shanghai 200030, P.R.China. Tel & Fax: +86 (0) 21 62822491 Email: yishi@sjtu.edu.cn (Yi Shi), xwli@sjtu.edu.cn (Xingwang Li) and heguangbiox@163.com (Guang He)

**Supplementary Table 1** Measurement scales

| Abbreviation | Scale | Scoring Scheme |
| --- | --- | --- |
| SWLS | Satisfaction With Life Scale | It includes 5 items in a 7-point response format ranging from 1(strongly disagree) to 7 (strongly agree). |
| PANAS | the Positive and Negative Affect Scale | It includes s 20 items on 5-point rating scale measuring positive affect score on items 1, 3, 5, 9, 10, 12, 14, 16, 17, 19 and negative affect score on items 2, 4, 6, 7, 8, 11, 13, 15, 18, 20;The sum up of positive and negative scores range from 10-50 respectively, with higher scores correspond to higher levels of positive or negative affect |
| OHQ | Oxford Happiness Questionnaire | It includes 8-item scale measuring self-rated happiness with the items on a 6-point Likert scale, ranging from 1 (strongly disagree) to 6 (strongly agree) |
| CES-D | Center for Epidemiologic Studies Depression Scale | It includes 20 questions reflecting 6 aspects of depression with a 4-point rating scale from ‘‘0’’ (never or few) to ‘‘3’’ (usually). The measurements are reversed for the 4th, 8th, 12th, and 16th items, i.e., from 0 to 5 meaning never or few. The sum up of all items range from 0 to 60, with the higher score correspond to more serious depression. |
| BFI | Big Five Inventory | It includes 44 items with 5-point Likert scale, ranging from 1 (strongly disagree) to 5 (strongly agree). The measurements of 16 items are reversed, i.e., from 1 (strongly agree) to 5 (strongly disagree). |

**Supplementary Table 2** R packages in this study

| R package | URL | Function |
| --- | --- | --- |
| psych | [https://cran.r-project.org/web/packages/psych](https://cran.r-project.org/web/packages/psych/) | principal component analysis |
| corrplot | <https://cran.r-project.org/web/packages/corrplot> | correlation analysis and its heat map |
| SNPassoc | https://cran.r-project.org/web/packages/SNPassoc | association analyses with five different genetic models: codominant, dominant, recessive, over-dominant and log-additive models |
| semMediation | <https://github.com/guhjy/semMediation> | analyzing the direct and indirect effects in multi-mediation models |
| mediation | <https://cran.r-project.org/web/packages/mediation> | analyzing the direct, indirect and total effects as well as percentage of mediating variables |

**Supplementary Table 3** Association between rs454214 and personality traits

| **Factors** | **Genotype rs454214** | **N** | **Mean** | **SE** | **β(95%CI)** | **p-value** |
| --- | --- | --- | --- | --- | --- | --- |
| Extraversion | **Codominant** |  |  |  |  |  |
|  | T/T | 279 | 25.86 | 0.39 | 0.00 | 0.0515 |
|  | T/C | 411 | 26.72 | 0.33 | -0.86(-0.16,1.88) |  |
|  | C/C | 184 | 25.37 | 0.52 | -0.49(-1.74,0.76) |  |
|  | **Dominant** |  |  |  |  |  |
|  | T/T | 279 | 25.86 | 0.39 | 0.00 | 0.3634 |
|  | T/C-C/C | 595 | 26.30 | 0.28 | 0.44(-0.51,1.40) |  |
|  | **Recessive** |  |  |  |  |  |
|  | T/T-T/C | 690 | 26.37 | 0.25 | 0.00 | 0.0733 |
|  | C/C | 184 | 25.37 | 0.52 | -1.00(2.09,0.09) |  |
|  | **Overdominant** |  |  |  |  |  |
|  | T/T-C/C | 463 | 25.66 | 0.31 | 0.00 | **0.0207^*^** |
|  | T/C | 411 | 26.72 | 0.33 | 1.05(0.16,1.95) |  |
|  | **log-Additive** |  |  |  |  |  |
|  | 0,1,2 |  |  |  | -0.13(-0.75,0.49) | 0.6709 |
| Agreeableness | **Codominant** |  |  |  |  |  |
|  | T/T | 279 | 34.62 | 0.34 | 0.00 | **0.0150^*^** |
|  | T/C | 411 | 35.64 | 0.26 | 1.02(0.18,1.86) |  |
|  | C/C | 184 | 34.47 | 0.42 | -0.15(-1.17,0.88) |  |
|  | **Dominant** |  |  |  |  |  |
|  | T/T | 279 | 34.62 | 0.34 | 0.00 | 0.1000 |
|  | T/C-C/C | 595 | 35.28 | 0.22 | 0.66(-0.13,1.45) |  |
|  | **Recessive** |  |  |  |  |  |
|  | T/T-T/C | 690 | 35.23 | 0.21 | 0.00 | 0.0996 |
|  | C/C | 184 | 34.47 | 0.42 | -0.76(1.66,0.14) |  |
|  | **Overdominant** |  |  |  |  |  |
|  | T/T-C/C | 463 | 34.56 | 0.26 | 0.00 | **0.0039^**^** |
|  | T/C | 411 | 35.64 | 0.26 | 1.08(0.35,1.81) |  |
|  | **log-Additive** |  |  |  |  |  |
|  | 0,1,2 |  |  |  | 0.03(-0.48,0.54) | 0.8945 |
| Conscientiousness | **Codominant** |  |  |  |  |  |
|  | T/T | 279 | 29.36 | 0.36 | 0.00 | 0.1087 |
|  | T/C | 411 | 30.17 | 0.30 | 0.81(-0.13,1.74) |  |
|  | C/C | 184 | 29.20 | 0.47 | -0.16(-1.30,0.98) |  |
|  | **Dominant** |  |  |  |  |  |
|  | T/T | 279 | 29.36 | 0.36 | 0.00 | 0.2559 |
|  | T/C-C/C | 595 | 29.87 | 0.26 | 0.51(-0.37,1.38) |  |
|  | **Recessive** |  |  |  |  |  |
|  | T/T-T/C | 690 | 29.84 | 0.23 | 0.00 | 0.2089 |
|  | C/C | 184 | 29.20 | 0.47 | -0.64(-1.64,0.36) |  |
|  | **Overdominant** |  |  |  |  |  |
|  | T/T-C/C | 463 | 29.30 | 0.28 | 0.00 | **0.0367^*^** |
|  | T/C | 411 | 30.17 | 0.30 | 0.87(0.06,1.68) |  |
|  | **log-Additive** |  |  |  |  |  |
|  | 0,1,2 |  |  |  | 0.01(-0.56,0.57) | 0.9807 |
| Neuroticism | **Codominant** |  |  |  |  |  |
|  | T/T | 279 | 23.60 | 0.33 | 0.00 | **0.0471^*^** |
|  | T/C | 411 | 22.87 | 0.31 | -0.73(-1.66,0.20) |  |
|  | C/C | 184 | 24.15 | 0.47 | 0.55(-0.59,1.69) |  |
|  | **Dominant** |  |  |  |  |  |
|  | T/T | 279 | 23.60 | 0.33 | 0.00 | 0.4535 |
|  | T/C-C/C | 595 | 23.27 | 0.26 | -0.33(-1.20,0.54) |  |
|  | **Recessive** |  |  |  |  |  |
|  | T/T-T/C | 690 | 23.17 | 0.23 | 0.00 | 0.0527 |
|  | C/C | 184 | 24.15 | 0.47 | 0.98(-0.01,1.98) |  |
|  | **Overdominant** |  |  |  |  |  |
|  | T/T-C/C | 463 | 23.82 | 0.27 | 0.00 | **0.0224^*^** |
|  | T/C | 411 | 22.87 | 0.31 | -0.95(-1.76,-0.14) |  |
|  | **log-Additive** |  |  |  |  |  |
|  | 0,1,2 |  |  |  | 0.18(-0.39,0.74) | 0.5412 |
| Openness to experience: | **Codominant** |  |  |  |  |  |
|  | T/T | 279 | 34.47 | 0.35 | 0.00 | 0.0726 |
|  | T/C | 411 | 35.28 | 0.31 | 0.82(-0.12,1.75) |  |
|  | C/C | 184 | 34.18 | 0.47 | -0.29(-1.43,0.86) |  |
|  | **Dominant** |  |  |  |  |  |
|  | T/T | 279 | 34.47 | 0.35 | 0.00 | 0.2857 |
|  | T/C-C/C | 595 | 34.94 | 0.26 | 0.48(-0.40,1.35) |  |
|  | **Recessive** |  |  |  |  |  |
|  | T/T-T/C | 690 | 34.95 | 0.23 | 0.00 | 0.1295 |
|  | C/C | 184 | 34.18 | 0.47 | -0.77(-1.77,0.23) |  |
|  | **Overdominant** |  |  |  |  |  |
|  | T/T-C/C | 463 | 34.35 | 0.28 | 0.00 | **0.0253^*^** |
|  | T/C | 411 | 35.28 | 0.31 | 0.93(0.12,1.75) |  |
|  | **log-Additive** |  |  |  |  |  |
|  | 0,1,2 |  |  |  | -0.05(-0.62,0.52) | 0.8673 |

Note. Significant P (<0.05) values are in bold .*p < 0.05; **p < 0.01; ***p < 0.001.

**Supplementary Table 4** Single mediation model test with bootstrapping

|  | Mediators | Neuroticism | | Agreeableness | | Openness | | Conscientiousness | | Extraversion | |
| --- | --- | --- | --- | --- | --- | --- | --- | --- | --- | --- | --- |
| **Y** |  | Estimate(95%CI) | p-value | Estimate(95%CI) | p-value | Estimate(95%CI) | p-value | Estimate(95%CI) | p-value | Estimate(95%CI) | p-value |
| Subjective Well-being | Indirect Effect | 0.16(0.03,0.29) | **0.016^*^** | 0.14（0.02，0.25） | **0.018^*^** | 0.11(0.00,0.21) | **0.05^*^** | 0.10(0.00,0.20) | 0.068 | 0.12(0.02,0.21) | **0.014^*^** |
|  | Direct Effect | 0.07(-0.04,0.17) | 0.24 | 0.08（-0.04，0.20） | 0.172 | 0.12(-0.02,0.27) | 0.096 | 0.13(-0.02,0.28) | 0.072 | 0.06(-0.03,0.14) | 0.194 |
|  | Total Effect | 0.23(0.06,0.40) | **0.008^**^** | 0.23（0.05，0.39） | **0.008^**^** | 0.23(0.06,0.41) | **0.008^**^** | 0.23(0.06,0.40) | **0.008^**^** | 0.18(0.04,0.32) | **0.008^**^** |
|  | Prop. Mediated | 0.71(0.25,1.34) | **0.02^*^** | 0.63（0.19，1.35） | **0.022^*^** | 0.46(0.03,1.18) | **0.05^*^** | 0.42(-0.04,1.15) | 0.072 | 0.67(0.22,1.39) | **0.014^*^** |
| Depressive symptoms | Indirect Effect | -1.72(3.14,-0.28) | **0.02^*^** | -1.43(-2.57,-0.27) | **0.02^*^** | -0.94(-1.85,0.05) | 0.064 | -0.92(-1.99,0.10) | 0.078 | 1.49(-2.84,-0.20) | **0.03^*^** |
|  | Direct Effect | -0.71(-1.97,0.46) | 0.242 | -1.00(-2.55,0.50) | 0.184 | -1.49(-3.21,0.27) | 0.108 | -1.51(-3.17,0.22) | 0.066 | -0.94(-2.37,0.44) | 0.218 |
|  | Total Effect | -2.43(-4.28,-0.80) | **0.01^**^** | -2.43,-4.31,-0.51) | **0.008^**^** | -2.43(-4.36,-0.29) | **0.018^*^** | -2.43(-4.27,-0.47) | **0.014^*^** | -2.43(-4.36,-0.56) | **0.004^**^** |
|  | Prop. Mediated | 0.71(0.21,1.46) | **0.01^*^** | 0.59(0.16,1.71) | **0.028^*^** | 0.39(-0.06,1.33) | 0.074 | 0.38(-0.11,1.13) | 0.084 | 0.61(0.16,1.45) | **0.03^*^** |

Note.

95%CI: 95% Confidence Interval.

Number of Bootstrap Resample: 1000.

Prop. Mediated: percentage of mediating variables explaining the association between rs454214 and DS/SWB


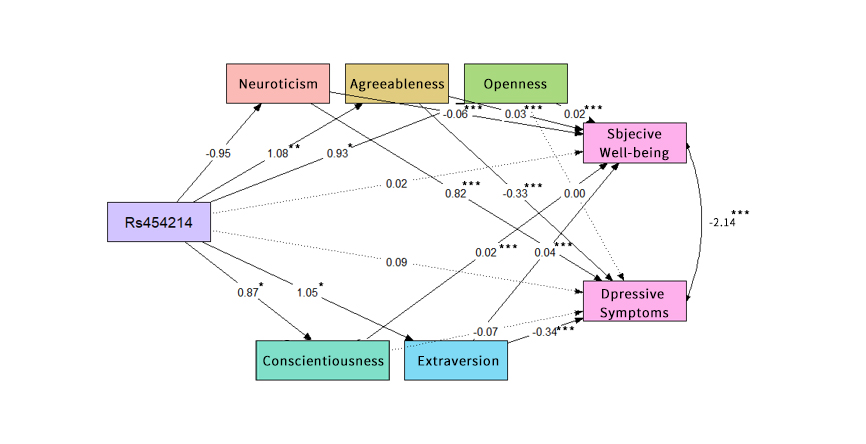


**Supplementary Figure 1.**

Multi-mediation plot shows estimates of the direct relations between rs454214, personality traits, subjective well-being and depressive symptoms. Full lines indicate signiﬁcant direct effects (p<0.05). Dashed lines indicate insigniﬁcant ones (p>0.05). *p < 0.05; **p < 0.01; ***p < 0.001.
